# Determinant factors influencing stunting prevention behaviors among working mothers in West Java Province, Indonesia: a cross-sectional study

**DOI:** 10.1186/s12889-025-24078-0

**Published:** 2025-08-09

**Authors:** Neti Juniarti, Ethar Alsharaydeh, Citra Windani Mambang Sari, Desy Indra Yani, Alison Hutton

**Affiliations:** 1 Department of Community Health Nursing, Faculty of Nursing, Universitas Padjadjaran, Bandung, Indonesia; 2 School of Nursing and Midwifery, Western Sydney University, Sydney, Australia; 3 South Western Sydney Local Health District, Liverpool, Sydney, Australia

**Keywords:** Stunting, Stunting prevention, Working mother, Work-related stress, Well-being

## Abstract

**Background:**

Stunting, a condition in which children fail to achieve their expected height for age, is a significant public health problem, particularly in low- and middle-income countries. The nutrition and caring practices of mothers and children during the first 1000 days of life determine the ability of a child to develop, learn, and thrive; however, mothers who work may not be able to ensure that their child is receiving adequate nutrition in the early stages of life. This study aimed to identify the determinants of working mothers’ stunting prevention behavior, including individual factors, work-related stress, well-being, knowledge, and attitudes toward the behavior of working mothers in supporting the prevention of stunting in West Java Province, Indonesia.

**Methods:**

This study used a cross-sectional design with a total sample of 225 working mothers in 78 offices mapped in Bandung city, Bekasi city, Bekasi Regency, Karawang Regency, West Bandung Regency, and Sumedang Regency in West Java Province, Indonesia. The inclusion criteria were as follows: working mothers aged 18 and above who reside and work in 6 cities/regencies of the study, have at least one child under the age of 5 years, and have consented to participate. The questionnaire consisted of demographic data and health characteristics, as well as questionnaires on knowledge, attitudes, work-related stress, well-being, and stunting prevention behavior. The data were analyzed using chi-square tests and logistic regression.

**Results:**

Among the 19 variables, two determinant factors influence stunting prevention behavior among working mothers in West Java Province, Indonesia. These factors are working mothers’ well-being (OR 3.30, *P* < 0.001) and knowledge about stunting prevention (OR 2.79, *P* < 0.001). A low level of well-being among working mothers increases the risk of poor stunting prevention behavior by 3.30 times. Similarly, insufficient knowledge about stunting prevention increases this risk by 2.79 times. The interplay between well-being and knowledge may improve mothers’ stunting prevention behavior.

**Conclusions:**

Working mothers’ well-being and knowledge of stunting prevention affect their stunting prevention behavior. This study is the first in Indonesia to explore the determinant factors of stunting prevention behavior among working mothers. Working mothers should receive special attention from the government regarding their rights to improve their well-being and knowledge related to their health. Further research should adopt longitudinal and intervention-based designs, include other caregivers, and explore diverse geographic and employment settings. Research on workplace and policy-level support systems is also recommended to inform more comprehensive strategies for improving child health outcomes.

**Supplementary Information:**

The online version contains supplementary material available at 10.1186/s12889-025-24078-0.

## Background

Stunting is a significant public health problem globally, particularly in low- and middle-income countries. Stunting refers to the impaired growth and development that children experience due to poor nutrition, repeated infection, and inadequate psychosocial stimulation [1]. It typically manifests as a low height for age, reflecting chronic malnutrition and illness during the early years of life, especially from conception to around the age of two [2]. The stunting rate in Indonesia decreased from 37.2% in 2013 [3] to 30.8% in 2018 [4]. The Indonesian government has set a target of 14% in 2024 for stunting prevalence [5]; however, this target is still out of reach, with a prevalence of stunting of 21.5% in 2023 [5]. Thus, this study aimed to identify the determinants of working mothers’ stunting prevention behavior.

Mothers who work may not be able to ensure that their child is receiving adequate nutrition in the early stages of life, as they may not be able to breastfeed or ensure that their child is adequately fed while they are at work [6]. The incidence of stunting among children varies significantly on the basis of maternal employment status, with some studies indicating higher rates among children of employed mothers (39.9–42.6%) [6, 7]. In contrast, others show the opposite (21.2% and 3.3%, respectively) [1, 2]. Stunting is a complex issue influenced by various socioeconomic and maternal factors, and research focusing on employed mothers is vital for developing targeted interventions and policies to reduce stunting rates among children from both employed and unemployed mothers. West Java Province is one of the provinces in Indonesia with a high stunting rate among under-fives, which is 30.9% in 2023 [4]. West Java Province is also one of the provinces with the largest number of female workers. West Java is the Indonesian province with the largest population. The total population of West Java in 2023 was 49.86 million, with 49.26% being female [8].

Additionally, a study showed that the Gini ratio of the maternal health index is unequal across the districts of West Java [9]. Therefore, West Java was chosen as the location for this study. The prevalence of stunting in West Java was 8.4% in 2022. The six districts selected as the study sites, based on their stunting prevalence rates, were West Bandung Regency (9.5%), Sumedang Regency (9.1%), Bekasi City (7.9%), Bandung City (7.7%), Bekasi Regency (4.3%), and Karawang Regency (2.7%) [10].

Child stunting in Indonesia is associated with the following determinants: male sex, premature birth, short birth length, nonexclusive breastfeeding for the first 6 months, short maternal height, low maternal education, low household socioeconomic status, living in a household with poor toilet hygiene and untreated drinking water, poor access to healthcare, and living in rural areas [11]. These determinants of stunting are related to the first 1000 days of life, from pregnancy until the first two years of life [12]. The nutrition and caring practices of mothers and children during the first 1000 days of life determine the ability of a child to develop, learn, and thrive. During this period, a child’s brain grows and develops, which is also the basis of the child’s long-term health [13].

The Government of Indonesia is strongly committed to reducing stunting. National policies such as Presidential Regulation Number 72/2021 and the Stunting Reduction Strategy (2021–2024) emphasize a multisectoral approach that includes support for working mothers. These policies recognize that working mothers face unique barriers—such as time constraints and limited access to health services—that can hinder optimal childcare practices. The government has taken decisive steps to accelerate nutritional improvement and stunting reduction, including nutrition-specific and nutrition-sensitive interventions through convergent, holistic, integrated, and high-quality activities jointly implemented by different sectors at the central, regional, and village levels [5]. One of the objectives of the stunting reduction program is to improve stunting prevention behaviors [5] that provide healthy eating, consistent, responsive interactions with caregivers, and caring settings [13]. Family function and family participation are needed to improve stunting prevention behaviors [14]; however, this effort may be disrupted when mothers work full-time. Working mothers face various challenges as they strive to balance their professional and personal lives.

Work‒life balance is often difficult for working mothers [15, 16]. Balancing work and family responsibilities can affect mothers’ physical health, mental well-being, and overall life satisfaction [17, 18]. Work-life balance can also affect how the family addresses health challenges and economic consequences and adapts related to maternal, newborn, and child health, as it can promote family resilience in the future [19].

In addition to working, women who work also have to perform their duties as housewives. Studies highlight the struggle to allocate time and energy effectively between professional commitments and caregiving duties [15]. This struggle may exacerbate fatigue and add a disproportionate burden to women. This burden can also have an adverse effect on pregnant women who work full-time. A study by Reynolds-Licciardello [20] showed that pregnant women lack the motivation to perform proper physical activity because they work full-time and care for other children. Owing to working full-time, women also have difficulty finding time to conduct regular antenatal check-ups and perform effective prenatal care to prevent maternal and infant mortality [21].

Pregnant women, nursing mothers, infants, and toddlers are vulnerable groups that need to be considered and protected so that their daily nutritional needs are met [22]. A study by Marangoni et al. [23] revealed that a balance of lifestyle and work was related to changes in the diet of pregnant women, especially in terms of consuming fruits and vegetables [23]. The change of diet is also the case for working women who are breastfeeding mothers. Poor nutritional fulfillment during pregnancy and breastfeeding can also increase the risk of stunting [11].

A complex interplay of social, economic, and personal factors influences stunting prevention behaviors among working mothers. Education, employment status, maternal characteristics [2, 24–27], and knowledge and perceptions of stunting risks [28–32] are significant determinants. Stunting prevention requires a comprehensive understanding of the multiple levels of influence that shape health behaviors. The ecological model [33] provides a framework for understanding how individual behavior is nested within interpersonal, organizational, community, and policy contexts. This ecological perspective supports the argument that interventions to reduce stunting should not focus solely on the mother’s behavior but rather address the interacting social and environmental systems that shape her decisions and capacities [33].

According to the health belief model, knowledge must be accompanied by perceived susceptibility, severity, benefits, and barriers to effectively influence behavior change [34]. Stunting prevention efforts focus on ensuring adequate nutrition and health care from the time a child is conceived through the first few years of life [35]. Prevention efforts are significant for working mothers because of the unique challenges they face in managing both their professional responsibilities and their children’s nutritional needs [36]. Mothers’ knowledge, attitudes, and behaviors significantly impact the prevention of stunting [37]. Various interventions are needed to strengthen multidisciplinary collaboration between stakeholders, including working mothers [11].

In Indonesia, since 2012, the Productive Healthy Worker Movement program has been implemented to encourage reproductive health efforts in the workplace to reduce maternal mortality, infant mortality, and stunting [38]. However, currently, there is no data on the status of stunting prevention behavior among working mothers, particularly mothers who work in the formal sectors in Indonesia. This study bridges this gap and highlights a specific group—working mothers—and examines how their well-being and knowledge influence their behavior toward preventing stunting. This study defines ‘working mothers’ as women aged 18 years and older who are actively engaged in the formal workforce while also managing their family responsibilities and are responsible for the care of at least one child. The age threshold aligns with Indonesia’s legal definition of adulthood and ensures ethical feasibility, as individuals under 18 are considered minors and face additional legal and social vulnerabilities. By focusing on working mothers in the formal sector, this study addresses an underexplored demographic whose behavior may differ due to their dual roles. This focus is significant, as a large proportion of stunting prevention efforts focus on general populations or traditional caregivers, such as homemakers. For this reason, a study of the determinant factors of stunting prevention behavior in working mothers is needed to reduce stunting in Indonesia.

## Methods

This study aimed to identify the determinants of working mothers’ stunting prevention behavior, including individual factors, work-related stress, well-being, knowledge, and attitudes toward the behavior of working mothers in supporting the prevention of stunting. This study used a cross-sectional design.

### Sample selection

This study targeted working mothers in the formal sectors within West Java Province, Indonesia. A purposive sampling strategy was used to select six out of the 27 city and district regions in the province: Bandung city, Bekasi city, Bekasi Regency, Karawang Regency, West Bandung Regency, and Sumedang Regency. These regions were chosen to represent diverse contexts within West Java, including the provincial capital and industrial zones, as well as suburban areas with a high concentration of working mothers in the formal sector.

Researchers sent formal letters of invitation to 171 offices across the selected regions. Of these, 78 offices granted permission for data collection. All working mothers in these formal sectors were approached directly by the researchers. The participants received an information sheet and were provided with details about the study before being invited to complete an online questionnaire. All the participants had internet access and literacy to complete the online questionnaire via their handphones or computers in their offices. Informed consent was obtained at the time of survey completion.

The inclusion criteria were as follows: (1) aged 18 years or older, (2) currently employed full-time in the formal (government or private) sector within one of the six selected areas, (3) had at least one child under the age of five, and (4) were willing to participate in the study. Women were excluded if they resided outside the designated areas, were unable to read, or could not complete the questionnaire in full.

The number of samples was determined using a sample calculation formula for the unknown population [39]. The samples were representative of working mothers in the formal sector. A total of 225 eligible working mothers consented to participate and were included in the final sample for analysis. The number of samples in Bandung city was 104, the number in Bekasi city was 29, the Bekasi Regency was 28, the Karawang Regency was 39, the West Bandung Regency was 21, and the Sumedang Regency was 4. All eligible respondents who completed the questionnaire were included. No respondents were excluded because of missing data or failure to meet the criteria.

### Study instruments

The study instrument consisted of 6 questionnaires as follows:

1. The knowledge on stunting questionnaire consists of 11 items with yes/no options. Correct answers were given a score of 2, and incorrect answers were given a score of 1. The cutoff point for categorizing knowledge level was a median score of 20.
2. The knowledge on stunting prevention questionnaire consists of 7 items with yes/no options. Correct answers were given a score of 2, and incorrect answers were given a score of 1. The cutoff point was the median score of 13.
3. The attitudes toward stunting questionnaire consists of 6 items rated from 1 (disagree) to 2 (agree). The cutoff point was the median score of 11.
4. The Stunting Prevention Behavior Questionnaire, which consists of 19 items with yes/no options, is scored as 1 (No) or 2 (Yes). All 19 items carry equal weights. The cutoff point for categorizing behavior was the median score of 35.

The reliability coefficient of the knowledge, attitude, and behavior questionnaires was 0.77 [40].

5. The WHO-5 Well-being Index, which is used to measure participants’ psychological well-being, consists of 5 items rated from 0 (at no time) to 5 (all the time) [41, 42]. The median score of 20 was used as the cutoff point to measure the respondents’ conditions, which consisted of 5 items ranging from 0 (at no time) to 5 (all the time). The Cronbach’s alpha of the Indonesian translation of the World Health Organization-5 Well-being Index (WHO-5) questionnaire was 0.853 [43].
6. The work stress questionnaire consists of 15 items on a Likert scale ranging from 1 (always) to 5 (never), adapted and translated from the 6th European Working Conditions Survey Questionnaire [44]. The cutoff point for categorizing stress levels was the median score of 57. The external quality assessment report was commissioned by Eurofound to the Tarki Social Research Institute and indicated that the fitness of the statistical output for the intended use remains high [45]. However, the report does not present a reliability coefficient.

The sociodemographic data collected included age, education level, and marital status. The occupational characteristics included employment status and job type.

The knowledge and behavior questionnaires were developed on the basis of the Indonesian government’s stunting reduction policy and reflect two categories of intervention: (1) nutrition-specific interventions (nine indicators) and (2) nutrition-sensitive interventions (eleven indicators) [3]. These questionnaires were reviewed by two experts in pediatric and maternity nursing. A pilot test was conducted with 20 respondents to assess readability. On the basis of feedback, eight questions were revised. Data from the pilot test were excluded from the primary analysis.

Data collection took place from August to November 2021. A combination of offline and online methods was used. Enumerators met participants face-to-face to ensure informed consent and distribute the online survey link, which was used to standardize responses and prevent duplicate entries.

### Data analysis

The data were analyzed using univariate, bivariate, and multivariate techniques. For univariate analysis, variables such as knowledge of stunting and its prevention, attitudes toward stunting, work-related stress, well-being, and stunting prevention behaviors were dichotomized into high and low categories on the basis of median cutoff points. Bivariate analysis was conducted using chi-square tests. Multivariate analysis was performed using logistic regression. In this study, potential confounding factors were addressed through multivariate logistic regression analysis. All independent variables that demonstrated a p-value of ≤ 0.25 in the bivariate analysis were included in the initial multivariate model. This threshold was chosen on the basis of established recommendations suggesting that a more liberal significance level at the bivariate stage helps ensure that potential confounders are not excluded too early in the modeling process [46]. This approach allows for better control of confounding effects and more accurate identification of variables independently associated with the outcome of interest.

The final model was developed using backward elimination, retaining variables that remained statistically significant at *p* < 0.05 while adjusting for the other covariates. This method enhances the robustness of the findings by accounting for interrelationships among variables and isolating the effects of key predictors. The regression model was evaluated using Wald tests, with p-values < 0.05 considered statistically significant. All analyses were conducted using IBM SPSS Statistics (Version 23) [47].

### Ethical consideration

This study obtained ethical approval from No. 795/UN6. KEP/EC/2021 in accordance with the Declaration of Helsinki. Involvement in this study was voluntary, and all participants provided consent to participate in this study. The researchers maintained the confidentiality of the data, and no identifying data was given to the employer.

## Results

Two hundred twenty-five women participated in this study. The average age of the women was 31 years, the highest level of education was university (81.9%), and most of the women were married (97.8%) (see Table 1).

**Table 1:** Descriptive summary of working mothers’ characteristics in West java, Indonesia (*n* = 225)

Variables	Frequency	Percentage
Age Mean: 31 years old (SD = 5.992)		
Education Level		
High School	43	19.1
University	182	81.9
Marital Status		
Married	220	97.8
Divorced/widow	5	2.2
Employment Status		
Permanent staff	171	76.0
Not permanent	54	24.0
Job Type		
Regular hours	206	91.6
Two shifts: morning and afternoon	19	8.4
State of Well-being Category		
Low	150	66.7
High	75	33.3
Stress Level Category		
High	110	48.9
Low	115	51.1
Had received information on stunting		
No	63	28.0
Yes	162	72.0
Sources of information on stunting		
Never received information	63	28.0
Health professionals	37	16.4
Internet	125	55.6
Exclusive Breastfeeding		
No	56	24.9
Yes	169	75.1

The occupational characteristics of the respondents revealed that most women were permanent staff (76%) and were working regular hours (91.6%). Most respondents had a low level of well-being (66.7%), and almost half of the respondents had a high level of work-related stress (48.9%). Most respondents received information on stunting (72%), and most of them obtained information from the internet (55%). Most respondents (75.1%) exclusively provide breastmilk to their children.

With respect to knowledge and attitudes, half of the respondents had good knowledge about stunting and its prevention (50.2% and 52.4%, respectively). Most of the respondents had favorable attitudes toward stunting prevention (75.6%) (see Table 2).

**Table 2:** Frequency distributions of knowledge, attitudes toward stunting, and working mothers’ behavior toward preventing stunting in West java, Indonesia (*n* = 225)

Variables	Frequency	Percentage
Knowledge about Stunting		
Low level of knowledge	112	49.8
High level of knowledge	113	50.2
Knowledge about stunting prevention		
Low level of knowledge	107	47.6
High level of knowledge	118	52.4
Attitude toward stunting prevention		
Not favorable	55	24.4
Favorable	170	75.6
Stunting Prevention Behavior		
Poor behavior	91	40.4
Good behavior	134	59.6

Overall, more than half of the working mothers reported good stunting prevention behavior (59.6%). A detailed description of the behavior of the participants is presented in Table 3.

**Table 3:** Descriptive items of working mothers’ behavior for stunting prevention in West Java, Indonesia (*n* = 225)

Item Questions	Percentage	
	Yes	No
Is your child eating regularly?	70.2	29.8
Does your child eat 3–5 times a day?	98.7	1.3
Do you give your child a snack?	100.0	0.0
Do you prepare food for your child yourself?	98.7	1.3
Does the food given to the child vary every day?	99.6	0.4
Do you wash dishes and glasses with running water and soap?	96.4	3.6
Do you bathe your child twice or more a day?	99.6	0.4
Does your child always wash their hands before eating?	81.8	18.2
Does your child always wash their hands after eating?	91.6	8.4
Do you wash your hands with soap after helping your child defecate?	94.2	5.8
Does your child wash their hands with soap after defecation?	86.2	13.8
Do you always wash your food before processing it?	80.9	19.1
Does your child wear footwear when playing outside?	72.9	27.1
Do you regularly clean your child’s nails?	99.6	0.4
Do you help your child brush their teeth more than 2 times a day?	100.0	0.0
Do you immediately give medicine to your child when they are sick?	86.2	13.8
Do you immediately take your child to the nearest health service when your child is sick?	55.4	44.6
Did you regularly check your pregnancy at the health service during pregnancy?	50.2	49.8
Was the mother assisted by a health worker when she gave birth?	65.9	34.1

The results revealed that of the 19 items, 7 were low. These were having regular check-ups during pregnancy (50.2%), taking the sick child to nearest health services (55.4%), using health professional birth attendance (65.9%), engaging in regular eating habits (70.2%), wearing footwear when playing outside (72.9%), washing food before processing (80.9%), hand washing before eating (81.8%), and taking medication for sick children (86.2%). These seven behaviors are essential for preventing stunting among children.

Of the 19 variables, six were correlated with working mothers’ behavior in terms of preventing stunting. These variables are income (OR 2.333, 95% CI [1.33–4.093], *P* = 0.003), monthly expenditure (OR 2.493, 95% CI [1.336–4.654], *P* = 0.004), state of well-being (OR 3.32, 95% CI [1.772–6.226], *P* < 0.001), work-related stress level (OR 2.194, 95% CI [1.275–3.778], *P* = 0.004), knowledge about stunting prevention (OR 2.816, 95% CI [1.624–4.884], *P* < 0.001) and attitudes toward stunting prevention (OR 2.144, 95% CI [1.158–3.972], *P* = 0.014) (Table 4).

**Table 4:** Factors correlated with working mothers’ behavior for stunting prevention in West java, Indonesia (*n* = 225)

Variables	Stunting Prevention Behavior		OR (95% CI)	*P*
	Less	Good		
Income per month from work			2.33	0.003
Below the minimum wage	42	36	[1.33–4.093]	
At and above the minimum wage	49	97		
Expenditure per month			2.49	0.004
Above the income	31	23	[1.336–4.654]	
At and below the income	60	111		
State of Well-being			3.32	< 0.001
Low	74	76	[1.772–6.226]	
High	17	58		
Work-related Stress Level			2.19	0.004
High	55	55	[1.275–3.778]	
Low	36	79		
Knowledge about stunting prevention			2.81	< 0.001
Low level	57	50	[1.624–4.884]	
High level	34	84		
Attitude toward stunting prevention			2.14	0.014
Not favorable	30	25	[1.158–3.972]	
Favorable	61	109		

Most potential confounding variables, such as mothers’ education, age, marital status, work status, job type, and source of stunting information, were not significantly correlated. However, logistic regression analysis highlighted two variables that are determinants of stunting prevention behavior, namely, mothers’ knowledge about stunting prevention and their well-being. After nonsignificant variables were removed, the final multivariate analysis results are presented in Table 5.

**Table 5:** Multivariate analysis of determinants of working mothers’ behavior for stunting prevention in West java, Indonesia (*n* = 225)

Variables	B	Exp (B)	Wald	95% CI for Exp (B)	*p* value
State of Well-being	1.19	3.30	13.141	1.730–6.293	0.000
Knowledge about stunting prevention	1.03	2.80	12.563	1.584–4.943	0.000
Constant	−2.71	0.07	18.446		0.000

**Fig. 1:**
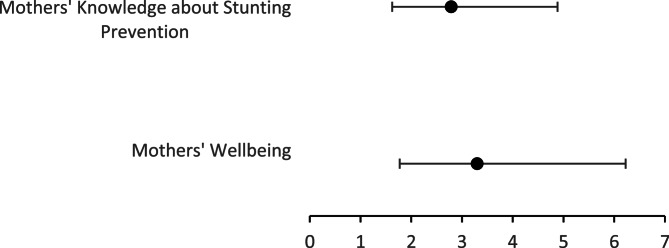
Odds ratios and 95% confidence intervals for the prevention of stunting

Table 5 shows that low well-being among working mothers increases the risk of poor stunting prevention behaviors by 3.30 times. Similarly, insufficient knowledge about stunting prevention increases this risk by 2.80 times. The strength and direction of these two significant predictors are presented in Fig. 1.

Table 5 shows that there are two determinant factors and that a constant should be incorporated into the mathematical equation as follows:

Y (Working mothers’ behavior for stunting prevention) = −2.71 + 1.19 × _1_ (State of Wellbeing) + 1.03 × _2_ (Knowledge about stunting prevention).

Using this mathematical equation, it can be inferred that when working mothers have a low level of well-being and a low level of knowledge, their behavior toward stunting prevention decreases by 2.71 times compared with that of mothers who have a high level of well-being and high level of knowledge about stunting prevention (*P* < 0.001).

## Discussion

The study revealed that well-being and knowledge about stunting prevention are key factors for working mothers’ behavior. This study revealed that of the 19 items of working mothers’ stunting prevention behavior, 2 were the lowest. These were having regular check-ups during pregnancy (50.2%) and taking the sick child to the nearest health services (55.4%). One of the most concerning results was the low percentage of working mothers attending regular check-ups during pregnancy (50.2%). Antenatal care (ANC) is essential for monitoring maternal and fetal health, reducing complications, and improving birth outcomes, as it can prevent the incidence of stunting [48, 49]. In many Indonesian communities, pregnancy is often perceived as a natural process that does not require medical intervention unless complications arise. This belief leads to a reliance on traditional birth attendants (TBAs) and a preference for home births over facility-based deliveries. A study analyzing national survey data revealed that mothers residing in areas with a high density of TBAs were significantly less likely to utilize maternal health services, including ANC and skilled birth attendance. Traditional practices were associated with lower odds of completing recommended maternal care services, even after controlling for socioeconomic factors [50].

Another concerning result was the low proportion of working mothers who were taking their sick child to the nearest health services (55.4%). Delayed healthcare-seeking behavior has been associated with increased child morbidity and mortality; for example, delayed healthcare-seeking behavior for diarrheal diseases can lead to complications and death [51]. The antibacterial immune function of 18-month-old children in a low-income setting is shaped by their stunting status and prior exposure to maternal inflammation [52]. Another study reported that caries in hypoplastic children with a history of neonatal developmental delay begins in the first year of life and may become more severe later in life, which may lead to chronic malnutrition [53].

The attitude toward stunting prevention significantly impacts stunting prevention behavior. This finding is similar to that of a study by Bimpong et al. [54], which reported that mothers’ attitudes toward infant and young child feeding practices were positive. Although the attitudes were positive, there were two difficulties perceived by the mothers, including difficulty in giving their children different types of food and difficulties in feeding their children several times a day, which impacted the actual behavior of the mothers [54]. Another study also revealed the importance of family care participation in children’s growth and development in West Java, Indonesia [13].

The positive association between well-being and stunting prevention behaviors aligns with other research findings on health behavior determinants. Previous research has consistently demonstrated that greater well-being, encompassing physical, emotional, and mental health, enhances individuals’ capacity to engage in preventive health behaviors [55]. For working mothers, who often juggle multiple roles, maintaining a high level of well-being may be crucial. The results of this study revealed that most working mothers were permanent staff (76%) and were working regular hours (91.6%). Thus, their family time was limited to before and after their work hours. Most of the mothers also experienced a high level of work-related stress (48.9%). The stress associated with balancing work and family responsibilities can impede their ability to focus on child health interventions [56].

Mothers’ subjective well-being (SWB), which includes feelings of happiness, meaningfulness, sadness, stress, and tiredness, plays a significant role in their parenting behavior [57]. Personal support is crucial for mothers’ well-being, which in turn affects their parenting roles [58]. Greater psychological well-being is associated with more positive parental attitudes, which can enhance the quality of mother‒child interactions [59]. Moreover, mothers’ life satisfaction is linked to more frequent shared activities with children, which positively affects children’s self-regulation and prosocial behavior [60].

The significant impact of knowledge on stunting prevention behavior corroborates findings from other health behavior studies, which underscore the importance of educational interventions [61]. The health belief model (HBM) suggests that knowledge must be accompanied by perceived susceptibility, severity, benefits, and barriers to effectively influence behavior change [34]. Mothers’ knowledge of stunting significantly shapes their perceptions of susceptibility and severity, which are core components of the HBM. Knowledge empowers mothers with the information necessary to make informed decisions about nutrition, hygiene, and critical healthcare practices to prevent stunting [11, 53]. A study revealed that mothers with higher knowledge levels perceived greater vulnerability and seriousness regarding stunting, leading to more proactive preventive behaviors [62]. Thus, educational interventions should be designed to enhance mothers’ understanding of the risks and consequences of stunting, the benefits of preventive measures, and ways to overcome potential barriers to implementing these measures.

The interplay between well-being and knowledge may improve mothers’ behavior. For example, a mother with high knowledge about stunting but low well-being may lack the energy or motivation to apply this knowledge effectively. Conversely, a mother with high well-being but low knowledge might be willing but unable to engage in stunting prevention behaviors. In Indonesian society, women bear heavy expectations from their families—husbands, children, and in-laws—regarding housekeeping and food preparation [63]. Working mothers may prepare all family meals daily before they go to work. When the family does not have a helper, working mothers may also wake up earlier and clean the house. These expectations may lead them to neglect their health and look after their family as tradition expects [63]. Studies show that for families that have a domestic helper, there is an ease in the day-to-day workload. Yet, it also comes with managerial responsibilities and may not entirely relieve mothers from household and childcare duties [64, 65].

Working mothers face multiple challenges in ensuring that their jobs are good and that their families are well served. The work-related stress level was significantly correlated with stunting prevention behavior (OR 2.194, 95% CI [1.275–3.778], *P* = 0.004). A lower level of schedule autonomy and psychological demands and a lack of perceived control in a stressful work environment negatively influence the mental health of women with young children [18]. Given these multiple burdens, mothers need an environment that supports their physical and mental health. Several strategies can be used to achieve work‒life balance in working mothers. Positive attributes, such as workplace flexibility in the form of flex-time (i.e., working from home or schedule flexibility), job-sharing, and working standard schedules, may be associated with reduced work‒family conflict and improved employee well-being [18]. The flexibility of time and workplace helps working mothers adjust their schedules to the rhythm of their daily activities. These findings highlight the importance of workplace policies that reduce stress and support working mothers in their dual roles.

The findings revealed a significant correlation between income and stunting prevention behavior (OR 2.333, 95% CI [1.33–4.093], *P* = 0.003). Higher income levels likely give mothers better access to nutritious food because the high cost is the primary factor affecting food choice and educational resources related to child nutrition and health [66]. The literature indicates that financial threats negatively correlate with job performance and positively correlate with perceived stress [67]. Monthly expenditures also strongly correlate with stunting prevention behaviors (OR 2.493, 95% CI [1.336–4.654], *P* = 0.004). Families also have other needs in addition to food, including clothing, health care, mobility, social relations, and education [68]. This monthly expenditure suggests that how families allocate financial resources can significantly impact children’s nutritional status. However, preparing and shopping for healthy food with a limited budget is not always feasible because of constraints such as a lack of time and energy, especially for full-time working parents and single-parent families [68]. Families with severe budget restrictions lead to food insecurity; families eat fewer fruits and vegetables and have a higher intake of cheap, energy-dense foods [69].

This study also identifies four other variables that significantly correlate with the behavior of working mothers in preventing stunting for their children. However, the four variables were not significant in the multivariate analysis. These variables are monthly income, monthly expenditure, work-related stress level, and attitudes toward stunting prevention. The variance inflation factor (VIF) for these four variables (1.032–1.152) indicates that no multicollinearity exists. It is also plausible that these four variables influence behavior indirectly through intermediate variables. Future studies may apply structural equation modeling (SEM) or mediation analysis to explore indirect effects.

The study revealed varied stunting prevention behaviors of the mothers, with the lowest engagement observed in antenatal care visits (50.2%) and seeking care for sick children (55.4%). These findings suggest that while some stunting prevention behaviors are well adopted, significant gaps remain in preventive and early intervention practices. To address these disparities, health policy should prioritize targeted interventions to increase awareness and accessibility of antenatal care and child health services. This policy includes strengthening community-based health education programs; enhancing the capacity and reach of primary healthcare services; and reducing financial, cultural, and logistical barriers to care. Additionally, since behaviors such as handwashing and food hygiene are relatively high, policies should aim to leverage these existing positive practices as entry points for broader health promotion strategies.

### Strengths and limitations

The strength of this study is that it is the first study in Indonesia to look at the determinant factors of stunting prevention behavior among working mothers. While this study provides valuable insights, it is not without limitations. The cross-sectional design precludes causal inferences, and future longitudinal studies are needed to establish causal relationships between well-being, knowledge, and stunting prevention behaviors. The questionnaire items are for general stunting prevention behaviors, not specifically for working mothers. Mothers also self-reported their knowledge, attitudes, or behaviors related to stunting prevention. Moreover, the study’s focus on working mothers may limit the generalizability of the findings to nonworking mothers, suggesting a need for broader research encompassing diverse parental roles and contexts. To mitigate these biases, researchers ensured a well-represented sample from 6 regions in West Java across 78 formal institutions, used validated measurement tools, and applied statistical controls for confounders.

## Conclusion

As a potential asset in economic growth in Indonesia, working mothers should receive special attention from the government regarding their rights and well-being. Given the associations among working mothers’ knowledge, attitudes, and preventive behaviors and targeted health education programs—especially in formal work environments—local governments and health authorities should prioritize the role of the mother in preventing stunting. Employers should be encouraged to implement family-friendly workplace policies, including flexible working arrangements, onsite child health education programs, and partnerships with local health services, to improve access to stunting-related information and care. Additionally, policies that address work-related stress and promote maternal well-being can further strengthen the capacity of working mothers to engage in effective child health practices. These measures align with Indonesia’s national strategy for reducing stunting and are essential for achieving sustainable improvements in early childhood nutrition and development.

It is recommended that the workplace provide health education on balanced nutrition and improving children’s health, as well as counseling for working mothers with infants under 2 years of age on the importance of regular child weight measurement and weighing. It is also important to advocate for office leaders about the importance of maintaining the well-being of working mothers through support and policies that can provide hybrid work models to help mothers balance work and family responsibilities. Additionally, programs that emphasize the importance of stunting prevention among working mothers should be developed so that the office can help mothers who have children under two years old monitor their children’s health regularly every month.

In addition to government and workplace policies, stunting prevention efforts require the empowerment of working mothers. By enhancing mothers’ self-efficacy, agency, and social support, reducing stunting programs can turn knowledge into action and transform structural access into lasting behavioral change. Empowered mothers are effective drivers of their children’s health outcomes.

Further research is needed to develop programs that can improve working mothers’ well-being and knowledge so that mothers can be supported to provide adequate nutrition, prevent child malnutrition, provide exclusive breastfeeding, provide adequate hygiene practices, and seek timely medical care for their children’s illnesses to prevent the incidence of stunting. Future research should also adopt longitudinal and intervention-based designs, include other caregivers, and explore diverse geographic and employment settings. Qualitative approaches and investigations into workplace and policy-level support systems are also recommended to inform more comprehensive strategies for improving child health outcomes through maternal empowerment.

## Supplementary Information

Supplementary Material 1.

## Data Availability

There could be ethical or legal constraints regarding the public sharing of our data. Researchers seeking access to our data should contact the corresponding author at neti.juniarti@unpad.ac.id.
