# Supplementary material for: Determinant factors influencing stunting prevention behaviors among working mothers in West Java Province, Indonesia: a cross-sectional study
Source: BMC Public Health. 2025 Aug 9;25:2719. doi: 10.1186/s12889-025-24078-0 (PMC12335024; doi:10.1186/s12889-025-24078-0)
Supplement: Supplementary file 1 — Supplementary Material 1. [file 12889_2025_24078_MOESM1_ESM.docx]

# Knowledge of stunting (11 questions)

| No. | Question | Answer | |
| --- | --- | --- | --- |
|  |  | Yes | No |
| 1 | Is a child’s growth primarily determined by the height and growth patterns of their parents? | 1 | 2 |
| 2 | Should toddlers get the full complement of basic immunizations (BCG, Hepatitis, Polio, DPT, and Measles) to increase their resistance to disease? | 2 | 1 |
| 3 | Are mothers who are malnourished early in pregnancy at risk of giving birth to short children? | 2 | 1 |
| 4 | Is monitoring the growth of children under five at the posyandu the first attempt to detect the occurrence of stunting? | 2 | 1 |
| 5 | Can inappropriate feeding patterns by parents be the cause of short toddlers? | 2 | 1 |
| 6 | Can a short toddler grow taller? | 2 | 1 |
| 7 | Should toddlers be brought to the posyandu every month for growth monitoring? | 2 | 1 |
| 8 | Is shortness more common in children aged 1-5 years? | 1 | 2 |
| 9 | Do short children have a lower intelligence level compared to other children? | 2 | 1 |
| 10 | Can delayed treatment in a stunted toddler lead to death? | 2 | 1 |
| 11 | Can a short toddler cause a toddler to be susceptible to disease? | 2 | 1 |

Scoring guide:

Correct answer 1, incorrect answer 0. All the answers are summed. The cut-off point for categorizing knowledge level was the median score of 20.[1]

1. **Knowledge about Stunting Prevention (7 questions)**

| No. | Question | Answer | |
| --- | --- | --- | --- |
|  |  | Yes | No |
| 1 | Is it possible to prevent stunting by providing nutritious food? | 2 | 1 |
| 2 | Is it possible to prevent stunting by exclusively breastfeeding? | 2 | 1 |
| 3 | Is it possible to prevent stunting by breastfeeding until 2 years of age? | 2 | 1 |
| 4 | Can the prevention of stunting be done by bringing children to health services when they are sick? | 2 | 1 |
| 5 | Is it possible to prevent stunting at the posyandu by providing supplementary food and milk? | 2 | 1 |
| 6 | Can full basic immunization (BCG, Hepatitis, Polio, DPT, and Measles) help prevent stunting in children? | 2 | 1 |
| 7 | Can counseling on health and nutrition at the posyandu for children under five help prevent stunting? | 2 | 1 |

Scoring guide:

Correct answers were scored 2, and incorrect answers were scored 1. The cut-off point was the median score of 13.[1]

1. **Attitude about Stunting (6 questions)**

| No. | Question | Answer | |
| --- | --- | --- | --- |
|  |  | Agree | Disagree |
| 1 | Malnutrition is the main nutritional problem in under-five years old | 2 | 1 |
| 2 | A long period of undernutrition due to feeding that does not meet nutritional needs result in a short toddler | 2 | 1 |
| 3 | Stunting or chronic malnutrition is another form of growth failure | 2 | 1 |
| 4 | A lack of nutritious and balanced food consumed by a toddler cause a toddler to be short | 2 | 1 |
| 5 | Children under five years old vulnerable to diseases caused by malnutrition | 2 | 1 |
| 6 | I belief non-exclusive breastfeeding a risk factor for a short toddler | 2 | 1 |

Scoring guide:

Attitudes towards Stunting questionnaire [1] consisting of 6 items rated from 1 (disagree) to 2 (agree). The cut-off point was the median score of 11.

1. **Stunting Prevention Behavior (19 questions)**

| No. | Question | Answer | |
| --- | --- | --- | --- |
|  |  | Yes | No |
| 1 | Is your child eating regularly? | 2 | 1 |
| 2 | Does your child eat 3-5 times a day? | 2 | 1 |
| 3 | Do you give your child a snack? | 2 | 1 |
| 4 | Do you prepare food for your child yourself? | 2 | 1 |
| 5 | Does the food given to the child vary every day? | 2 | 1 |
| 6 | Do you wash dishes and glasses with running water and soap? | 2 | 1 |
| 7 | Do you bathe your child more than twice a day? | 2 | 1 |
| 8 | Does your child always wash their hands before eating? | 2 | 1 |
| 9 | Does your child always wash their hands after eating? | 2 | 1 |
| 10 | Do you wash your hands with soap after helping your child defecate? | 2 | 1 |
| 11 | Does your child wash his/her hands with soap after defecation? | 2 | 1 |
| 12 | Do you always wash your food before processing it? | 2 | 1 |
| 13 | Does your child wear footwear when playing outside? | 2 | 1 |
| 14 | Do you regularly clean your child's nails? | 2 | 1 |
| 15 | Do you help your child brush their teeth more than 2 times a day? | 2 | 1 |
| 16 | Do you immediately give medicine to your child when they are sick? | 2 | 1 |
| 17 | Do you immediately take your child to the nearest health service when your child is sick? | 2 | 1 |
| 18 | Did you regularly check your pregnancy at the health service during pregnancy? | 2 | 1 |
| 19 | Was the mother assisted by a health worker when she gave birth? | 2 | 1 |

Scoring guide:

Stunting prevention behavior questionnaire [1], consisting of 19 items with yes/no options, scored as 1 (No) and 2 (Yes). All 19 items carry equal weight. The cut-off point for categorizing behavior was the median score of 35.

1. **A summary table linking items to nutrition-specific and nutrition-sensitive domains** in the stunting prevention behavior questionnaire is provided in the supplementary material.

| Stunting Prevention Domains | Number in Questionnaire |
| --- | --- |
| Nutrition specific | 1, 2, 3, 4, 5, 16, 17, 18, 19 |
| Nutrition sensitive | 6, 7, 8, 9, 10, 11, 12, 13, 14, 15 |

1. **WHO-5 Well-being Index [2, 3]**

| **Please respond to each item by marking one box per row, regarding how you felt in the last two weeks** | | **All the time (5)** | **Most of the time (4)** | **More than half the time (3)** | **Less then half the time (2)** | **Some of the time (1)** | **At no time (0)** |
| --- | --- | --- | --- | --- | --- | --- | --- |
| WHO 1 | I have felt cheerful in good spirit |  |  |  |  |  |  |
| WHO 2 | I have felt calm and relaxed |  |  |  |  |  |  |
| WHO 3. | I have felt active and vigorous |  |  |  |  |  |  |
| WHO 4. | I woke up feeling fresh and rested |  |  |  |  |  |  |
| WHO 5. | My daily life has been filled with things that interest me |  |  |  |  |  |  |

Scoring guide:

WHO-5 Well-being Index, used to measure participants’ psychological well-being, consisting of 5 items rated from 0 (at no time) to 5 (all the time) [2, 3]. The median score of 20 was used as the cut-off point. to measure the participants conditions, consisted of 5 items ranging from 0 (at no time) to 5 (all the time).

1. **Work-related Stress Questionnaire from the 6th European Working Conditions Survey Questionnaire 2015 [4]**

|  | | **Always (1)** | **Most of the time (2)** | **Sometimes (3)** | **Rarely (4)** | **Never (5)** |
| --- | --- | --- | --- | --- | --- | --- |
| 1. | Your colleagues help and support you |  |  |  |  |  |
| 2 | Your manager helps and support you |  |  |  |  |  |
| 3. | You are consulted before objectives are set for your work |  |  |  |  |  |
| 4. | You are involved in improving the work organisation or work process of your department or organisation |  |  |  |  |  |
| 5. | You have a say in the choice of your work colleagues |  |  |  |  |  |
| 6. | You can take a break when you wish |  |  |  |  |  |
| 7. | You have enough time to get the job done |  |  |  |  |  |
| 8. | Your job gives you the feeling of work well done |  |  |  |  |  |
| 9. | You are able to apply your own ideas in your work |  |  |  |  |  |
| 10. | You have the feeling of doing useful work |  |  |  |  |  |
| 11. | You know what is expected of you at work |  |  |  |  |  |
| 12. | You are treated fairly at your workplace |  |  |  |  |  |
| 13. | You experience stress in your work |  |  |  |  |  |
| 14. | You can influence decision that are important for your work |  |  |  |  |  |
| 15. | Your jobs required that you hide your feeling |  |  |  |  |  |

Scoring guide:

Work stress questionnaire, consisting of 15 items on a Likert scale ranging from 1 (always) to 5 (never), adapted and translated from the 6th European Working Conditions Survey Questionnaire [2]. The cut-off point for categorizing stress levels was the median score of 57.

Reference:

1. Rahayuwati L, Nurhidayah I, Hidayati NO, Hendrawati S, Agustina HS, Ekawati R, Setiawan ASJEJoB: **Analysis of factors affecting the prevalence of stunting on children under five years**. 2020, **14**(2).

2. Sischka PE, Costa AP, Steffgen G, Schmidt AFJJoADR: **The WHO-5 well-being index–validation based on item response theory and the analysis of measurement invariance across 35 countries**. 2020, **1**:100020.

3. Topp CW, Østergaard SD, Søndergaard S, Bech PJP, psychosomatics: **The WHO-5 Well-Being Index: a systematic review of the literature**. 2015, **84**(3):167-176.

4. European Foundation for the Improvement of Living and Working Conditions: **6th European Working Conditions Survey (2015) Questionnaire**. In*.* Dublin, Ireland: European Foundation for the Improvement of Living and Working Conditions; 2015.
